# Papillary Carcinoma Within a Thyroglossal Duct Cyst

**DOI:** 10.7759/cureus.74407

**Published:** 2024-11-25

**Authors:** Vishva S Shah, Rahul U Vaidya, Shashank C Desai, Jaimish P Gajjar

**Affiliations:** 1 General Surgery, Gujarat Cancer Society (GCS) Medical College Hospital and Research Centre, Ahmedabad, IND

**Keywords:** papillary carcinoma, sistrunk procedure, tgdc, thyroglossal duct cyst, total thyroidectomy

## Abstract

Thyroglossal duct cysts (TGDCs) are typically located in the midline of the neck. Carcinomas arising within these cysts are extremely rare, with papillary carcinoma being the most common type. Diagnosis is generally confirmed postoperatively following excision. We report the case of a 38-year-old female diagnosed with papillary carcinoma within a TGDC who underwent a Sistrunk procedure followed by a total thyroidectomy.

## Introduction

Thyroglossal duct cysts (TGDCs) are congenital anomalies arising from remnants of the thyroglossal duct [1]. They occur in approximately 7% of the adult population and typically present as a painless midline neck mass, often located below the hyoid bone [2]. Although TGDCs are benign, there is a rare potential for malignant transformation, predominantly to papillary carcinoma [1]. Here we present a rare case of papillary carcinoma arising from a TGDC, highlighting the diagnostic challenges and the management strategy.

A TGDC is formed because of failure of closure of the thyroglossal duct, which extends from the foramen caecum at the base of the tongue up to the thyroid in the neck and is an embryological remnant [3]. The thyroglossal duct usually involutes by the end of the tenth week of gestation, and if any part of the duct remains persistent, secretions from the epithelial layer will cause inflammation and lead to the formation of a TGDC [4]. 

## Case presentation

**Figure 1:**
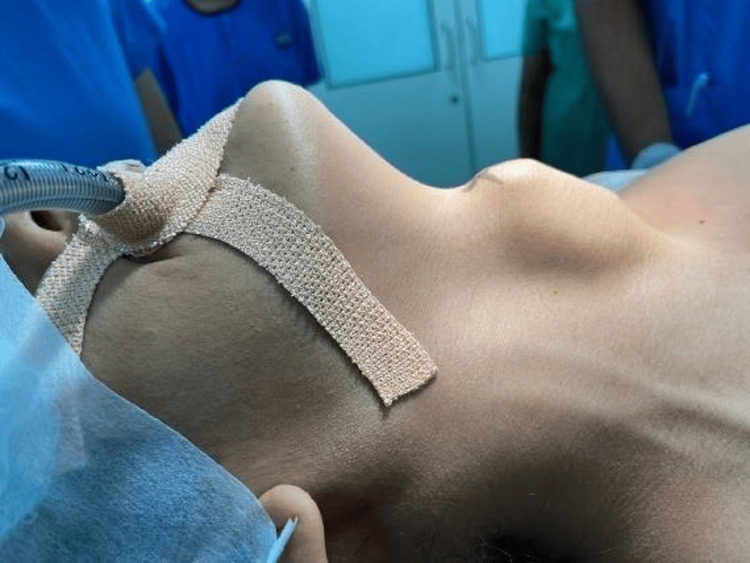
Clinical picture of the patient

**Figure 2:**
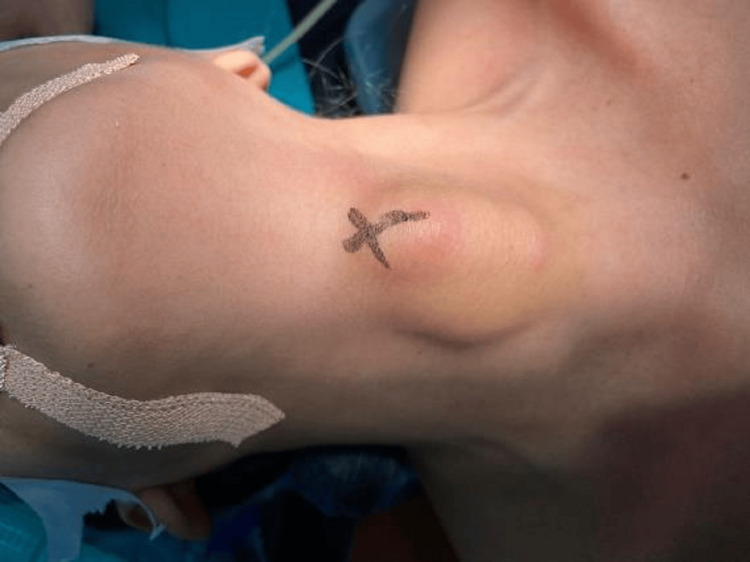
Clinical picture of the patient

A 38-year-old female presented with a three-month history of progressively enlarging midline neck mass measuring approximately 3 cm x 2 cm. The mass was asymptomatic. Examination revealed a soft, mobile mass in the midline of the neck just below the hyoid bone, which moved with swallowing and had minimal movement on tongue protrusion. The patient's thyroid function tests were within normal limits. No regional lymphadenopathy was noted. Clinical pictures are shown in Figures 1-2.

Investigations

**Figure 3:**
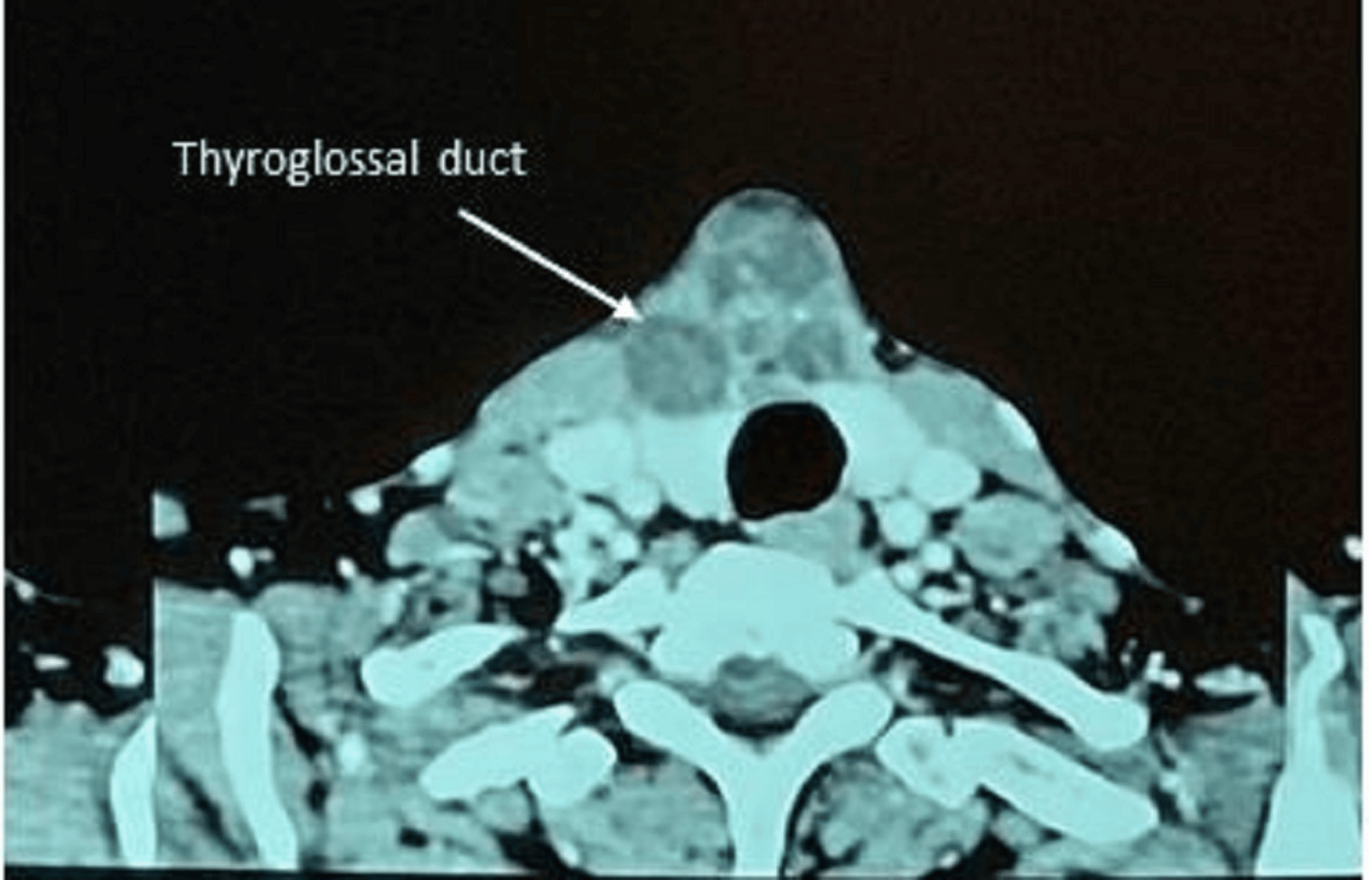
CT scan images (axial section)

**Figure 4:**
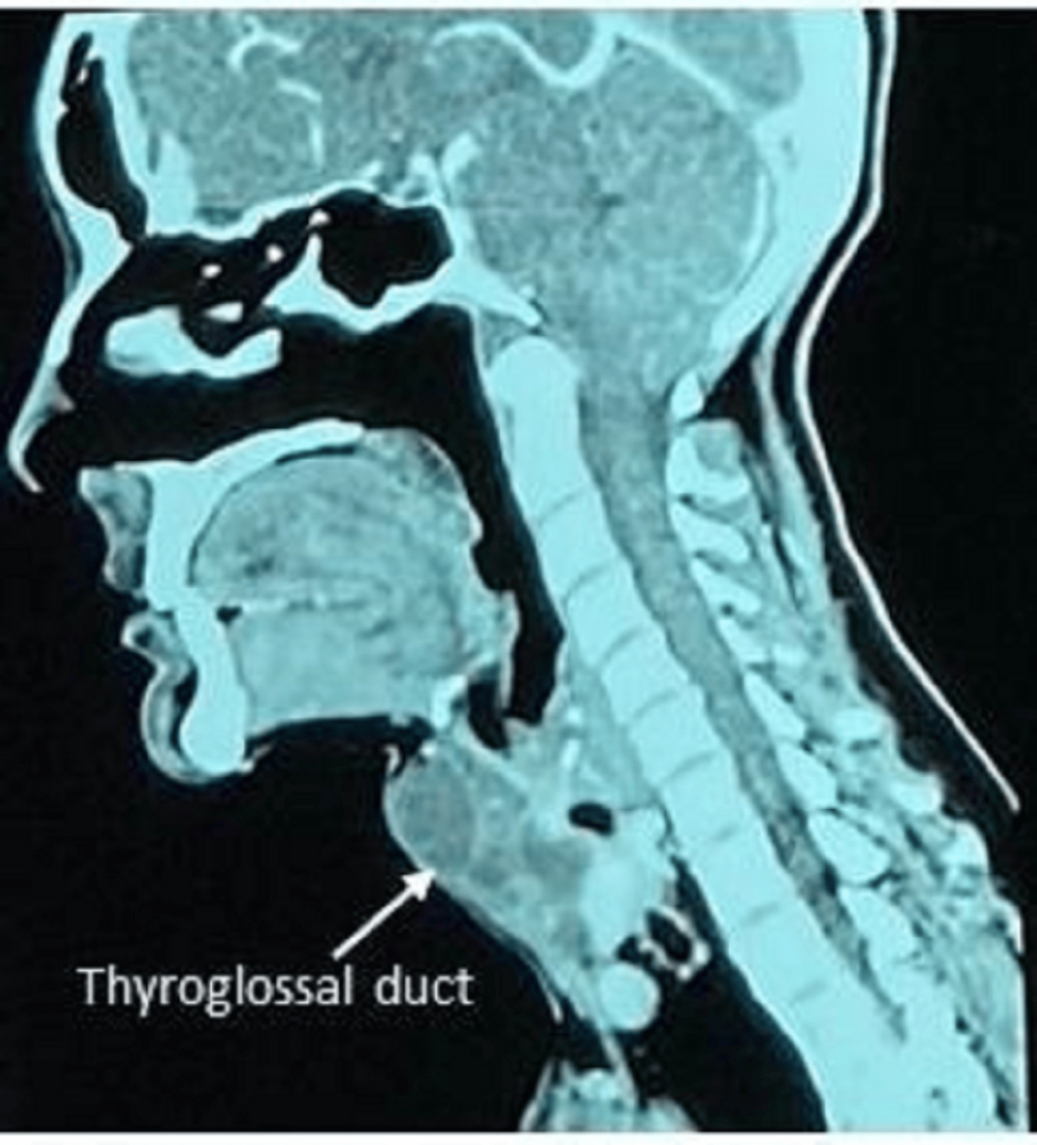
CT scan (sagittal section)

Ultrasound and CT scan showed a well-defined, predominantly cystic lesion measuring approximately 33 mm x 38 mm x 40 mm, which was identified at the midline above the isthmus of the thyroid. The lesion was close to both thyroid lobes, and there was loss of fat plane with the isthmus. Multiple enhancing septations and foci of calcification were noted. The CT scan images are shown in Figures 3-4.

Fine-needle aspiration cytology (FNAC) yielded straw-coloured fluid. Microscopic examination showed cystic macrophages, squamous cells, and an inflammatory background suggestive of a benign cystic lesion.

Treatment

The patient underwent a Sistrunk procedure, where the TGDC tract along with surrounding tissue was dissected above up to the foramen caecum, and also the part of the hyoid bone was excised.

Postoperative histopathology revealed a cyst wall lined by cuboidal epithelium with mass in the thyroglossal duct. The cyst revealed complex branching, randomly oriented papillae with fibrovascular cores associated with thyroid follicles and psammoma bodies. The papillae contained a central core of fibrovascular tissue, lined by one or occasionally several layers of cuboidal cells with crowded-over nuclei. The nuclei were overlapping with finely dispersed chromatin, and micronuclei with areas of fibrosis. The diagnosis was suggestive of papillary carcinoma in the thyroglossal duct cyst.

**Figure 5:**
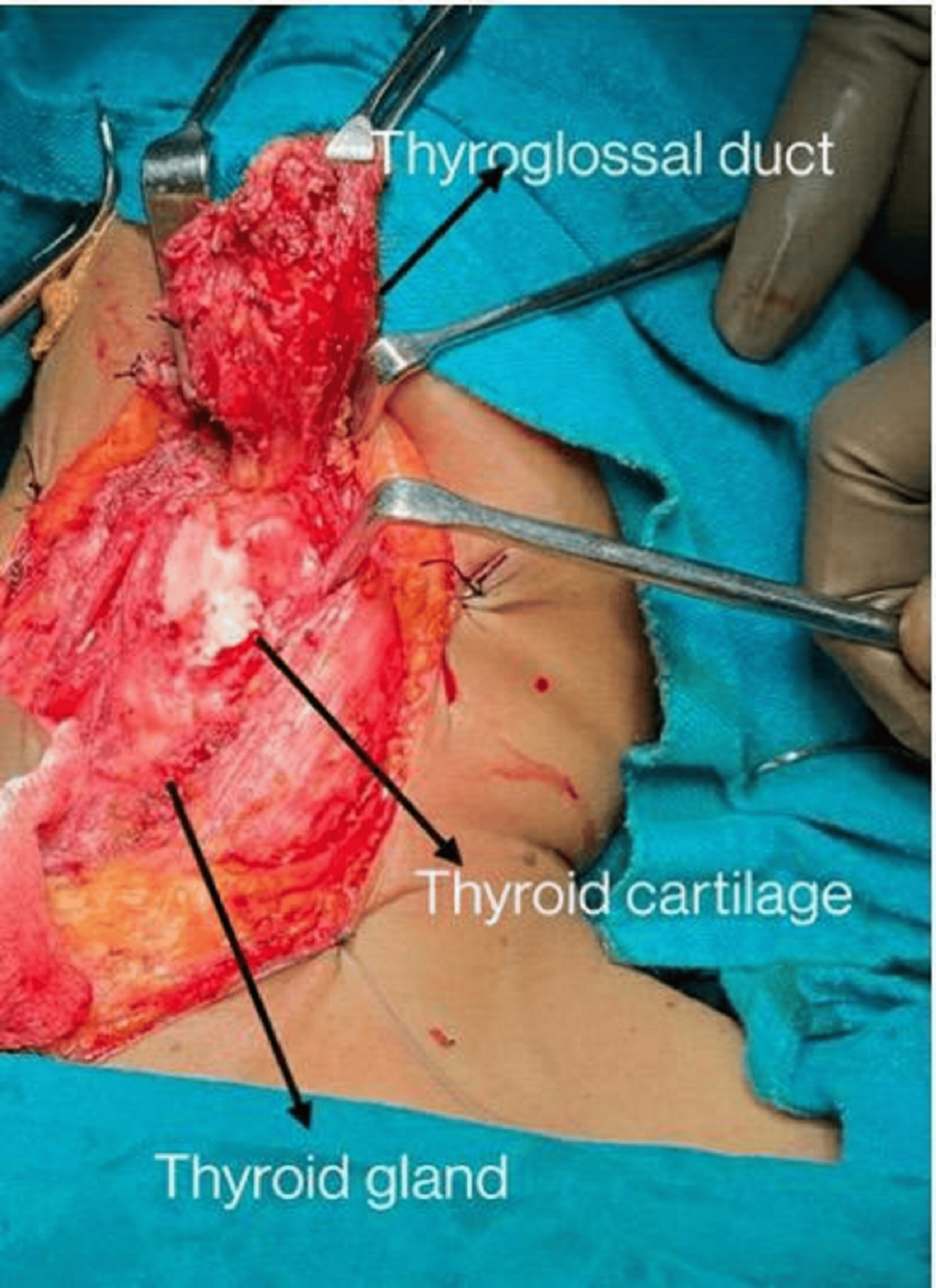
Intraoperative picture of the thyroglossal duct cyst

**Figure 6:**
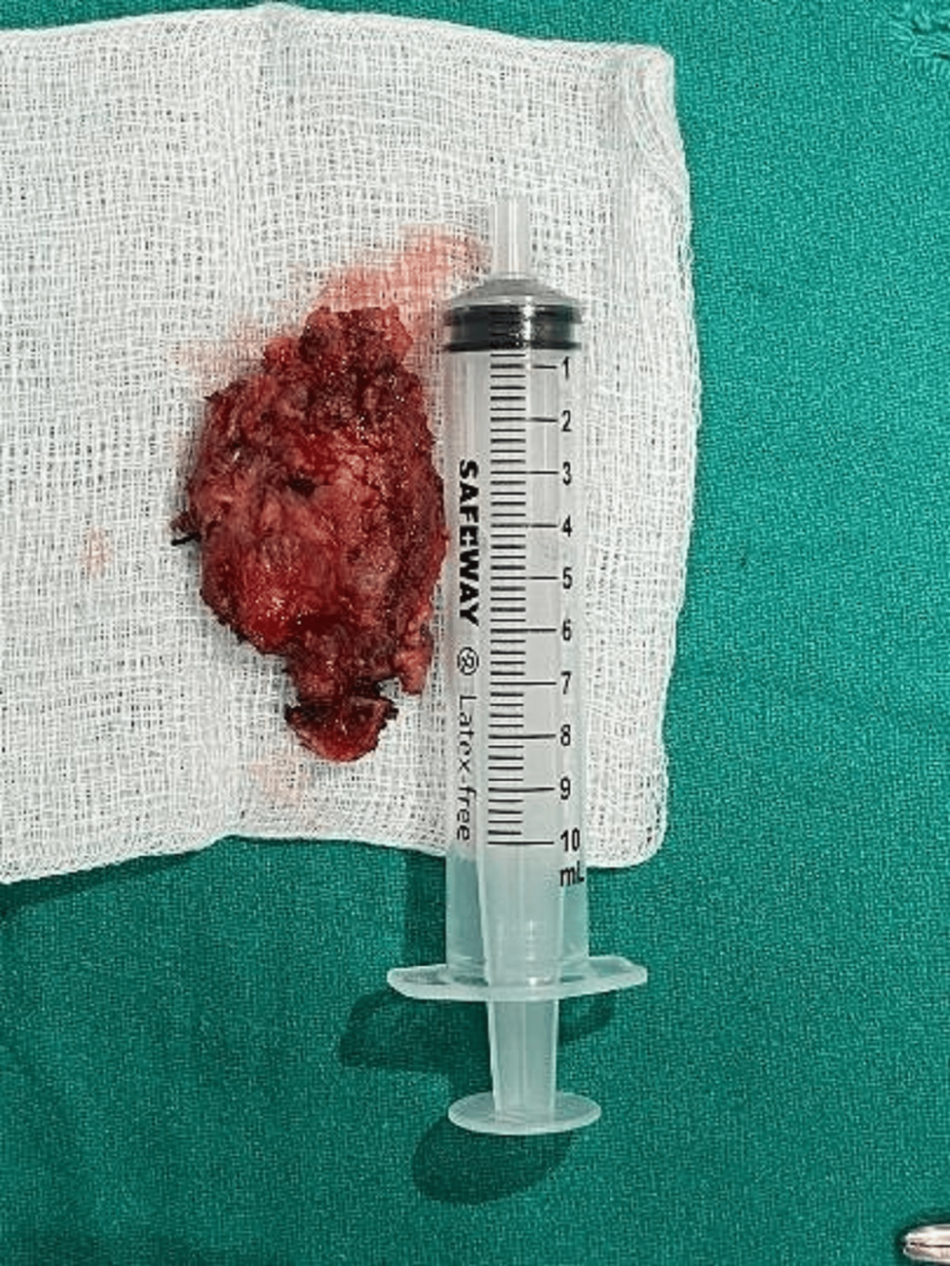
Excised specimen of the thyroglossal duct cyst

**Figure 7:**
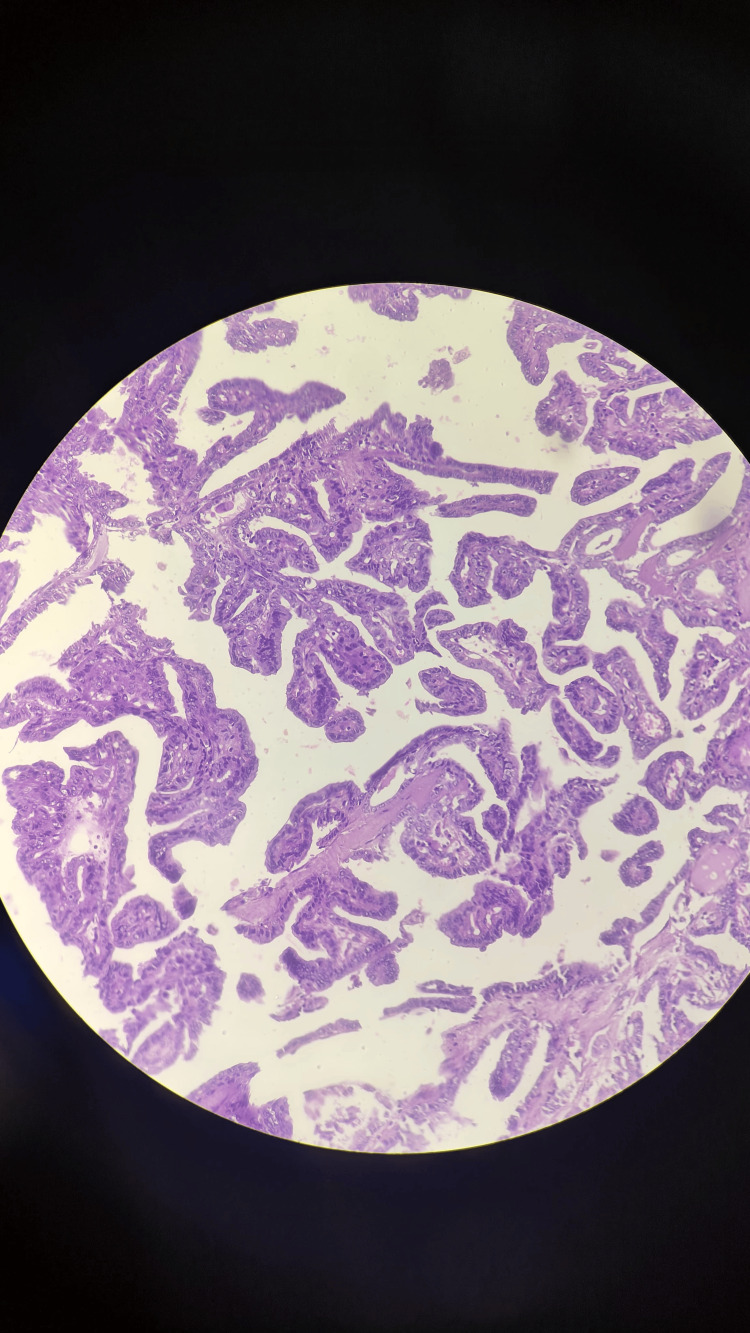
Histopathological (microscopic) image of the papillary carcinoma within the thyroglossal duct cyst

The intraoperative picture and excised specimen are shown in Figures 5-6, respectively. A histopathological (microscopic) image of papillary carcinoma within the thyroglossal duct cyst is shown in Figure 7.

**Figure 8:**
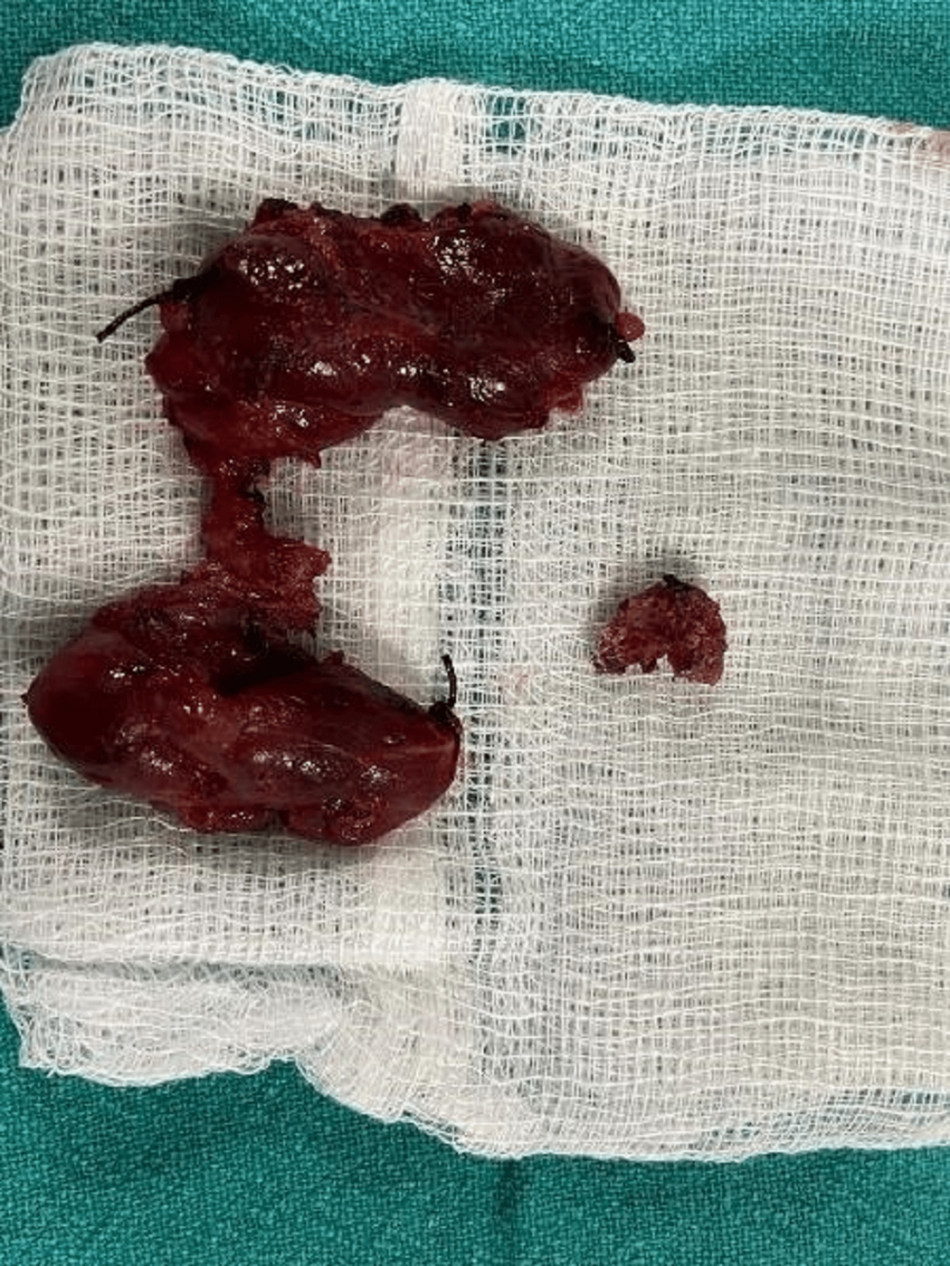
The excised specimen after a total thyroidectomy

Based on the reports of histopathology of the excision of the TGDC, a total thyroidectomy was performed for the patient one week after the previous surgery. The postoperative histopathological report revealed that sections from the thyroid showed normal histology of the thyroid with congestion. There was no atypia or granuloma. Figure 8 shows an excised specimen of total thyroidectomy.

## Discussion

Thyroglossal duct cyst carcinomas are exceedingly rare, accounting for less than 1% of all thyroid malignancies [5]. Papillary carcinoma is the most common histological subtype, making up 94% of cases, while squamous cell carcinoma is much rarer. The pathogenesis is controversial, with theories suggesting either ectopic thyroid tissue within the cyst or metastasis from an occult thyroid carcinoma [6]. 

Diagnosis is often made incidentally by histopathological reports of the gross specimen following surgical excision, as clinical and radiological findings rarely indicate malignancy preoperatively. In our case, FNAC did not reveal malignancy, and the diagnosis was confirmed postoperatively.

The management of papillary carcinoma arising in a TGDC remains controversial. While the Sistrunk procedure, which is the removal of the TGDC, the middle part of the hyoid bone, and surrounding tissue around the thyroglossal duct tract, is generally considered adequate for benign TGDC, total thyroidectomy is recommended for cases with malignant transformation, especially if there is evidence of invasion or when the tumour exceeds one centimetre in size [7]. In this case, a total thyroidectomy was performed following a diagnosis of papillary carcinoma, and postoperative recovery was uneventful. The patient is currently under regular six-monthly follow-up to look for recurrence, if any.

## Conclusions

Thyroglossal duct cyst carcinoma is a rare entity, often diagnosed postoperatively. Imaging and FNAC are important in the preoperative evaluation of neck masses, though malignancy may only be identified with the histopathological report following surgery. In cases of papillary carcinoma, total thyroidectomy in addition to the Sistrunk procedure is recommended to prevent recurrence and manage the risk of metastasis. Long-term follow-up is crucial due to the risk of recurrence.
